# Effects of Genicular Nerve Blocks in Combination With an Adductor Canal Block in Patients Undergoing Arthroscopic Knee Surgery: A Randomized Controlled Trial

**DOI:** 10.7759/cureus.79215

**Published:** 2025-02-18

**Authors:** Poramed Amorntodsapornpong, Kornkanok Yuwapattanawong, Shinichi Sakura

**Affiliations:** 1 Department of Anesthesiology, Faculty of Medicine, Vajira Hospital, Navamindradhiraj University, Bangkok, THA; 2 Department of Anesthesiology, Unnan Municipal Hospital, Shimane University, Izumo, JPN

**Keywords:** adductor canal block, analgesia, genicular nerve blocks, knee arthroscopic surgery, opioid consumption, peripheral nerve blocks, postoperative pain

## Abstract

Background and objectives: Arthroscopic knee surgery is increasingly popular. Optimal postoperative pain management enhances patient satisfaction and minimizes hospitalization. Numerous studies have demonstrated the benefits of adductor canal blocks (ACBs) and genicular nerve blocks (GNBs) in postoperative analgesia. This study aims to evaluate the efficacy of adding GNBs to an ACB in reducing postoperative pain scores compared to an ACB alone. Additionally, it seeks to compare secondary outcomes, including opioid consumption, motor blockade, nausea and vomiting, rash, and itching during the postoperative period.

Method: This prospective, randomized, controlled clinical trial included 49 patients undergoing arthroscopic knee surgery. The study group received a GNB with 0.25% bupivacaine (3 mL) at each quadrant of the knee, except for the inferolateral quadrant, in combination with an ACB using 0.25% bupivacaine (20 mL). The control group received an ACB alone. Fentanyl (1-2 mcg/kg IV) was administered as rescue analgesia during the perioperative period. Pain scores (visual analog score (VAS)), cumulative opioid consumption, motor blockade, and incidences of postoperative nausea and vomiting (PONV) and itching were assessed at six, 12, 24, and 48 hours postoperatively.

Results: No statistically significant differences in median pain scores were observed between the groups. However, postoperative opioid consumption was significantly lower in the study group, with median values (interquartile range (IQR)) of 0 (0, 30) at 12, 24, and 48 hours postoperatively compared to the control group (p ≤ 0.001). Additionally, no significant differences were found between groups regarding motor blockade or opioid-related side effects.

Conclusion: Adding GNBs to an ACB did not demonstrate superiority in reducing postoperative pain scores. However, it effectively reduced perioperative opioid consumption at 12 to 48 hours postoperatively without increasing adverse effects such as nausea, vomiting, or motor blockade. These findings highlight the potential of GNBs as a valuable component of postoperative pain management strategies for arthroscopic knee surgery.

## Introduction

In recent years, there has been a growing preference for performing arthroscopic surgery in patients with knee injuries, as it offers several advantages, including smaller incisions, reduced tissue trauma, faster recovery, and lower complication rates compared to traditional open surgery [1]. However, despite these benefits, post-surgical pain remains a significant concern, as it can delay early mobilization, hinder participation in physical therapy, and ultimately prolong the overall recovery process. Studies have shown that inadequate pain control following knee surgery is associated with impaired functional outcomes, decreased patient satisfaction, and an increased risk of developing chronic pain syndromes. Several factors have been identified as potential contributors to postoperative pain intensity. These include the duration of the surgical procedure, the specific characteristics of the intervention, the duration of tourniquet application (if used), and the severity of preoperative pain. Patients with higher preoperative pain levels often report greater postoperative pain intensity, suggesting that preoperative pain management strategies may also play a role in optimizing recovery [2,3].

Currently, multimodal analgesia is widely adopted to enhance postoperative pain relief while minimizing opioid consumption and its associated side effects, such as nausea, vomiting, sedation, respiratory depression, and the risk of dependency. Among the various analgesic techniques available, the adductor canal block (ACB) has emerged as an effective strategy for pain management following knee surgery. By selectively anesthetizing the saphenous nerve and providing sensory blockade to the anteromedial aspect of the knee, the ACB has been shown to significantly reduce pain levels, lower opioid requirements, and improve postoperative functional outcomes. Importantly, an ACB is preferred over a femoral nerve block (FNB) in many cases, as it provides pain relief while preserving quadriceps muscle strength, thereby facilitating early mobilization and rehabilitation [4-7].

In addition to ACBs, genicular nerve blocks (GNBs) have gained attention as a promising adjunct for pain control in knee surgery. The genicular nerves, which include the superomedial, superolateral, and inferomedial branches, play a crucial role in innervating the knee joint. Studies have demonstrated that combining a GNB with an ACB in total knee arthroplasty (TKA) results in superior pain relief, greater opioid-sparing effects, and improved overall recovery compared to ACB alone. This combined approach provides a more comprehensive sensory blockade of the knee joint, effectively targeting both the anterior and posterior innervation pathways [8-10].

However, despite the growing body of evidence supporting the efficacy of GNBs in TKA, there is a notable lack of research specifically evaluating their role in arthroscopic knee surgery. Given that arthroscopic procedures, while less invasive than TKA, still involve significant tissue manipulation, it is essential to explore whether the addition of GNBs to an ACB can further enhance postoperative pain control, reduce opioid consumption, and improve functional recovery in this patient population.

Therefore, the objective of our study is to assess the potential benefits of incorporating GNBs into the standard ACB protocol for patients undergoing arthroscopic knee surgery. We hypothesize that the addition of GNBs will result in lower postoperative pain scores and reduced opioid requirements compared toan ACB alone, as the combined technique will provide more comprehensive coverage of the knee joint’s innervation. If proven effective, this approach could help refine postoperative pain management strategies, enhance patient recovery, and reduce reliance on opioid medications, ultimately improving clinical outcomes for patients undergoing arthroscopic knee procedures.

## Materials and methods

Study design

A comprehensive two-arm, parallel, prospective triple-blind (interventionist, patients, and assessor) randomized controlled trial was conducted to investigate and compare the efficacy of two anesthetic techniques: the combination of GNBs with an ACB versus an ACB alone. Two groups of patients undergoing knee arthroscopic procedures for meniscus repair and anterior cruciate ligament (ACL)/posterior cruciate ligament (PCL) repair or debridement, excluding reconstruction, were compared. All participants were recruited with a consent form at Vajira Hospital, Navamindradhiraj University, Bangkok, Thailand. The Institutional Review Board (IRB) of the Faculty of Medicine Vajira Hospital approved the protocol on August 13, 2023 (approval number: COA 138/2566). The trial was registered with the Thai Clinical Trials Registry (TCTR) on April 11, 2024 (registration number: TCTR 20240411003).

Participants

Patients who scheduled knee arthroscopic procedures for meniscus repair and ACL/PCL repair or debridement after writing the informed consent and receiving subarachnoid anesthesia at Vajira Hospital Navamindradhiraj University, Bangkok, Thailand, from September 10, 2023, to April 10, 202,4 were enrolled. The same surgical team performed each surgical intervention. Inclusion criteria were as follows: elective unilateral knee arthroscopy for repairing the meniscus, ACL/PCL repair or debridement, age 18 to 80 years, and American Society of Anesthesiologists (ASA) physical status of I to III. The exclusion criteria were as follows: patient’s refusal to do peripheral nerve blocks, cognitive inability, pre-existing joint-related disease, chronic pain of more than six months, allergic to opioids, nonsteroidal anti-inflammatory drugs (NSAIDs), sulfa, or local anesthetic drugs, and contraindicated for local injection. Upon identification of eligible participants, the research team initiated contact and provided detailed guidance on the research protocol. Subsequently, the process involved obtaining informed consent, enrolling patients, and implementing randomization procedures.

Sample size calculation

The minimum required sample size per group, factoring in a 10% dropout rate, was determined to be 21 patients. This calculation was derived from the formula presented in [11-12]. The sample size and power calculations formula is as follows:

$\mathrm{n}_{1}^{}=\frac{\mathrm{\left( \mathrm{}_{}^{}\mathrm{Z}_{\mathrm{1-\frac{\alpha}{2}}}+Z\mathrm{}_{1-\beta}^{}\mathrm{}_{}^{}\right)}_{}^{2}\left[\mathrm{\sigma}_{1}^{2}+\frac{\mathrm{\mathrm{\sigma}_{2}^{2}}_{}^{}}{\frac{\mathrm{n}_{1}^{}}{\mathrm{n}_{2}^{}}}\right]}{\mathrm{\left( \mathrm{\mu}_{1}^{} -\mathrm{\mu}_{2}^{}\right)}^{2}}$

where Ratio (r) of 1.00, an alpha (α) of 0.05, Z(0.975) of 1.959964, beta (β) of 0.200, and Z(0.800) of 0.841621.

Our study expected the difference in pain scores at six hours post operation, which was our primary outcome between the two groups because it is the timing that loses the effect of spinal anesthesia. According to a study by Kukreja et al. (2021) [13], the mean postoperative pain score at six hours in group one was 1.54 (μ₁), with a standard deviation (σ₁) of 2.74. In the comparison group, the mean postoperative pain score at six hours was 4.15 (μ₂), and the standard deviation (σ₂) was 2.95.

Randomization, allocation concealment, and blinding

Following the inclusion, a block-of-four randomization was executed utilizing a computer-generated number list. An independent researcher strictly prepared the randomization by creating blocks for groups A and B with a block size of four. The blocks were then randomly ordered using software, and participants were sequentially assigned to the groups. Through this process, individuals were randomly allocated to either receive a combination of GNBs with an ACB (study group) or an ACB alone (control group). The allocation information was securely enclosed within opaque, sealed envelopes, with each envelope designated for delivery to a specialized anesthesiologist in charge of peripheral nerve blocks upon the participant's entrance into the operating room. Ensuring a comprehensive blinding protocol, the orthopedist, patient, and assessor remained unaware of the specific anesthetic technique employed. The orthopedist was only permitted access to information about the peripheral nerve block after its completion.

Interventions

The interventions in this study comprised a specialized anesthesiologist administering a combination of GNBs with an ACB. The intervention protocol unfolded in three distinct phases. Initially, upon the patient's entry into the operating room, a shared practice for both groups involved standard monitoring (blood pressure, pulse rate, real-time electrocardiogram, and oxygen saturation). Subsequently, intravenous midazolam injection for 1-2 mg and fentanyl 0.5-1 mcg/kg was administered for sedation, supplemented with 2 L/min of oxygen via an oxygen cannula. Following this, subarachnoid anesthesia was conducted using 0.5% hyperbaric bupivacaine (3 ml) with the patient in lateral positioning, and a sensory test was performed to assess coverage of at least the T10 dermatome level. Notably, every patient in this protocol received care from the same anesthesiologist and orthopedist.

The second phase of the study involved the implementation of distinct anesthetic techniques for peripheral nerve blocks. In the study group, an approach was employed: the administration of GNBs with 0.25% bupivacaine (3 ml) at each specific location targeting the superomedial, superolateral, and inferomedial branches of the genicular nerve. This was integrated with an ACB, where 0.25% bupivacaine (20 ml) was applied. In contrast, the control group underwent a more streamlined procedure, exclusively receiving an ACB with 0.25% bupivacaine (20 ml). By using a 3-20 MHz linear transducer ultrasonography (GE VENUE GO, GE HealthCare, Chicago, IL, USA), providing optimal visualization for the administration of the GNBs, the 22G needle with a length of 1.5 inches was used to perform GNBs. Simultaneously, for the ACB, a size 22G needle with a length of 80 millimeters was utilized.

The third phase was the administration of 4 mg of dexamethasone after the peripheral nerve block was performed and 4 mg of ondansetron before skin closure for postoperative nausea and vomiting prophylaxis. Additionally, for sedation purposes, propofol was infused intravenously at a rate of 25 to 100 mcg/kg/min, with adjustments made based on the desired level of sedation and the patient's vital signs. A standardized approach was implemented for managing intraoperative conditions. For instances of intraoperative hypotension and bradycardia, ephedrine at doses of 5 mg to 10 mg intravenously and atropine at 0.6 mg intravenously were prescribed. Postoperatively, the pain management protocol involved the administration of parecoxib 40 mg intravenously every 12 hours for two days. Additionally, fentanyl at a dose of 1 to 2 mcg/kg/dose intravenously was provided for breakthrough pain relief as needed.

Data collection

Sociodemographic and clinical information was collected before surgery, while surgical and anesthetic details were acquired during and after the procedure. Standard intraoperative monitoring, such as noninvasive blood pressure (NIBP), peripheral oxygen saturation (SpO₂), and heart rate (HR), was documented every five minutes. Intraoperative data recording, encompassing fentanyl dose, total propofol infusion dose, ephedrine dose, midazolam dose, peripheral nerve blocking time (referring to the duration from the initiation of the peripheral nerve block procedure until its completion), total anesthetic time (encompassing the period from the initiation of spinal anesthesia to the completion of the orthopedic procedure), and tourniquet time, was conducted immediately following surgery completion. The postoperative period followed up at six hours (T6) and continued at 12 hours (T12), 24 hours (T24), and 48 hours (T48) in the patient's room.

Outcome measures

*Primary Outcom*es

The primary outcome of this research was to assess the efficacy of adding GNBs to an ACB, comparing it to the use of an ACB alone in patients undergoing arthroscopic knee surgery by only one anesthesiologist who was blind to the intervention. Effectiveness was assessed through a comprehensive analysis of postoperative pain scores and the supplementary requirements for analgesic drugs. The assessment of pain utilized the visual analog scale (VAS), ranging from 0 to 100, by only one assessor who was blinded. Data were collected at distinct time intervals, specifically at six hours postoperatively, according to the outcomes that we referred to to calculate the sample size.

Secondary Outcomes

The secondary outcomes were pain utilizing the VAS and postoperative opioid consumption at 12, 24, and 48 hours post operation and postoperative opioid consumption at six, 12, 24, and 48 hours post operation. Additionally, the incidence of side effects, including local anesthetic systemic toxicity (LAST), motor blockade assessed through quadriceps muscle strength using straight leg raising for motor grade V, ankle movement evaluated via dorsiflexion for motor grade V, and the occurrence of postoperative nausea, vomiting, and itching, will be evaluated.

## Results

**Figure 1:**
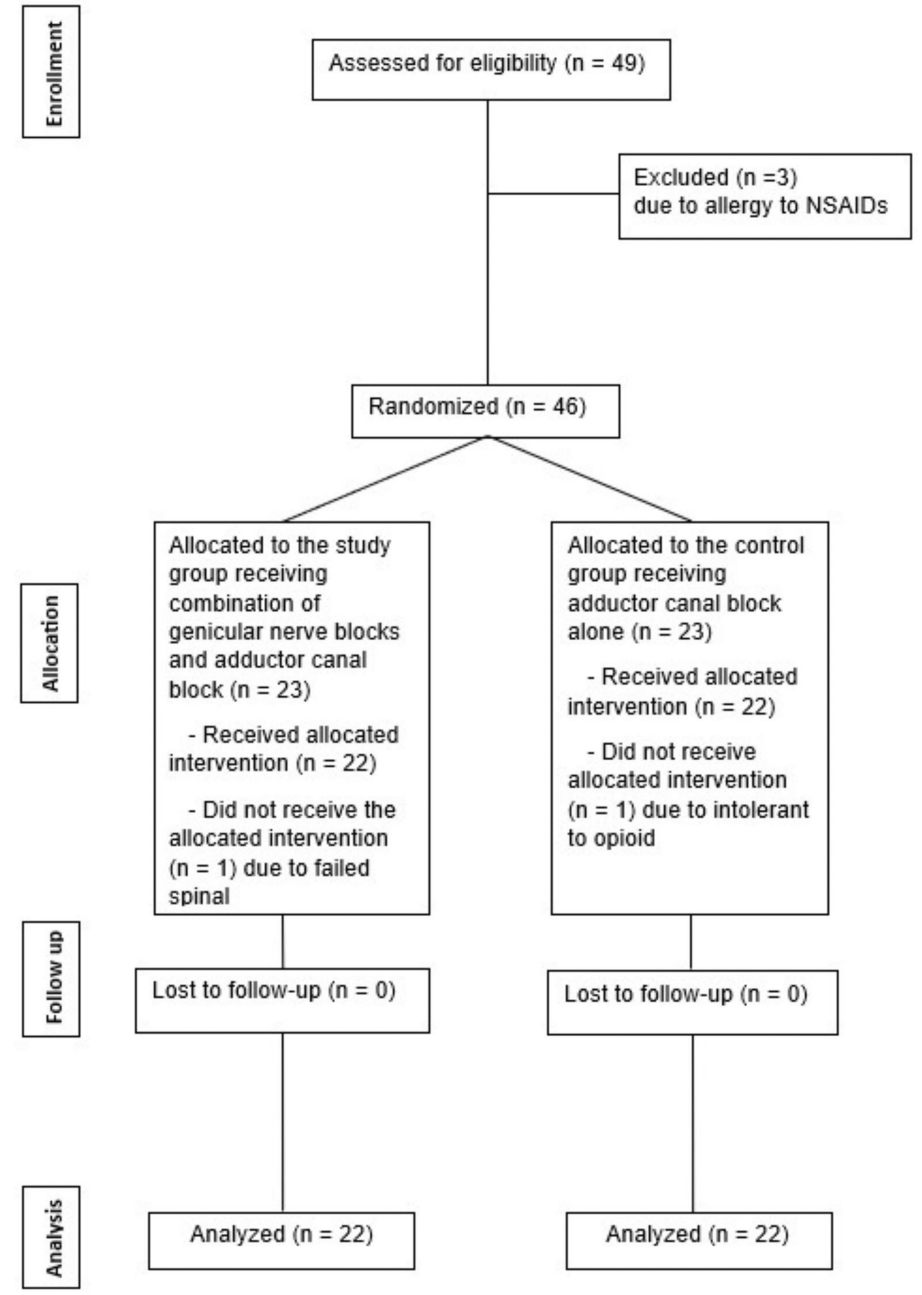
A CONSORT diagram shows the flow of each stage of the randomized controlled trial CONSORT: Consolidated Standards of Reporting Trials; NSAIDs: nonsteroidal anti-inflammatory drugs

Forty-nine patients scheduled for knee arthroscopic surgery met the eligibility criteria for participation in this study and were subsequently randomly assigned into two groups (Figure 1). However, only 44 patients, comprising 22 individuals in each group, completed the study protocol and were consequently subjected to data analysis. Notably, three subjects were excluded from the study due to reported allergic reactions to NSAIDs presenting with skin rash from the history. Furthermore, one participant from the initial group was omitted from the analysis due to failed spinal anesthesia, while another individual from the second group was excluded owing to intolerance to opioid side effects with excessive dizziness, nausea, and vomiting.

In the analysis conducted, no statistically significant differences were observed between the groups concerning variables such as age, sex, body mass index, underlying medical conditions, type of surgical procedure, total anesthetic duration, tourniquet application duration, baseline pain score, or intraoperative administration of fentanyl, propofol, ephedrine, and midazolam. However, there was a statistically significant disparity in peripheral nerve block duration between the two groups (p=0.007) (Table 1).

**Table 1:** Sociodemographic and clinical profiles of the participants Patients in the study group received GNBs with an ACB and those in the control group received only an ACB. Chi-square test and independent t-test or Mann-Whitey U test; A p-value <0.05 was considered significant. n: total number of subjects in each group; BMI: body mass index; ASA: American Society of Anesthesiologists; ACL: anterior cruciate ligament; PCL: posterior cruciate ligament; SD: standard deviation; IQR: interquartile range; ACB: adductor canal block; GNBs: genicular nerve blocks

Patient and surgical character	Study group (n=22)	Control group (n=22)	p-value
Age, mean ± SD (years)	42.3 ± 16.7	40.9 ± 13.7	0.762
Sex, number of female participants (%)	14 (63.6%)	11 (50%)	0.361
BMI, mean ± SD (kg/m^2^)	24.18 ± 5.69	26.75 ± 5.43	0.133
ASA grade (%)			0.948
ASA I	8 (36.4%)	9 (40.9%)	-
ASA II	11 (50%)	10 (45.5%)	-
ASA III	3 (13.6%)	3 (13.6%)	-
Surgical type, n (%)			
Medial meniscus repair	13 (59.1%)	13 (59.1%)	1
Lateral meniscus repair	1 (4.5%)	4 (18%)	0.154
ACL repair	6 (27.3%)	4 (18%)	0.472
PCL repair	5 (22.5%)	6 (27.3%)	0.728
Intraoperative time (min), mean ± SD			
Blocking time (min)	5.7 ± 1.9	4.4 ± 0.9	0.007*
Anesthetic time (min)	157.5 ± 34.4	149.1 ± 40.5	0.462
Tourniquet time (min)	98.6 ± 29.7	94.1 ± 35.2	0.645
Intraoperative drugs, mean ± SD			
Fentanyl (mcg)	51.1 ± 12.1	54.6 ± 16.6	0.441
Propofol (mg), median (IQR)	250 (200, 500)	300 (140, 460)	0.878
Ephedrine (mg), median (IQR)	12 (6, 12)	2 (0, 12)	0.062
Midazolam (mg), median (IQR)	2 (2, 2)	2 (1, 2)	0.937
Preoperative pain, median (IQR)	15 (0, 44)	26 (0, 50)	0.611

**Figure 2:**
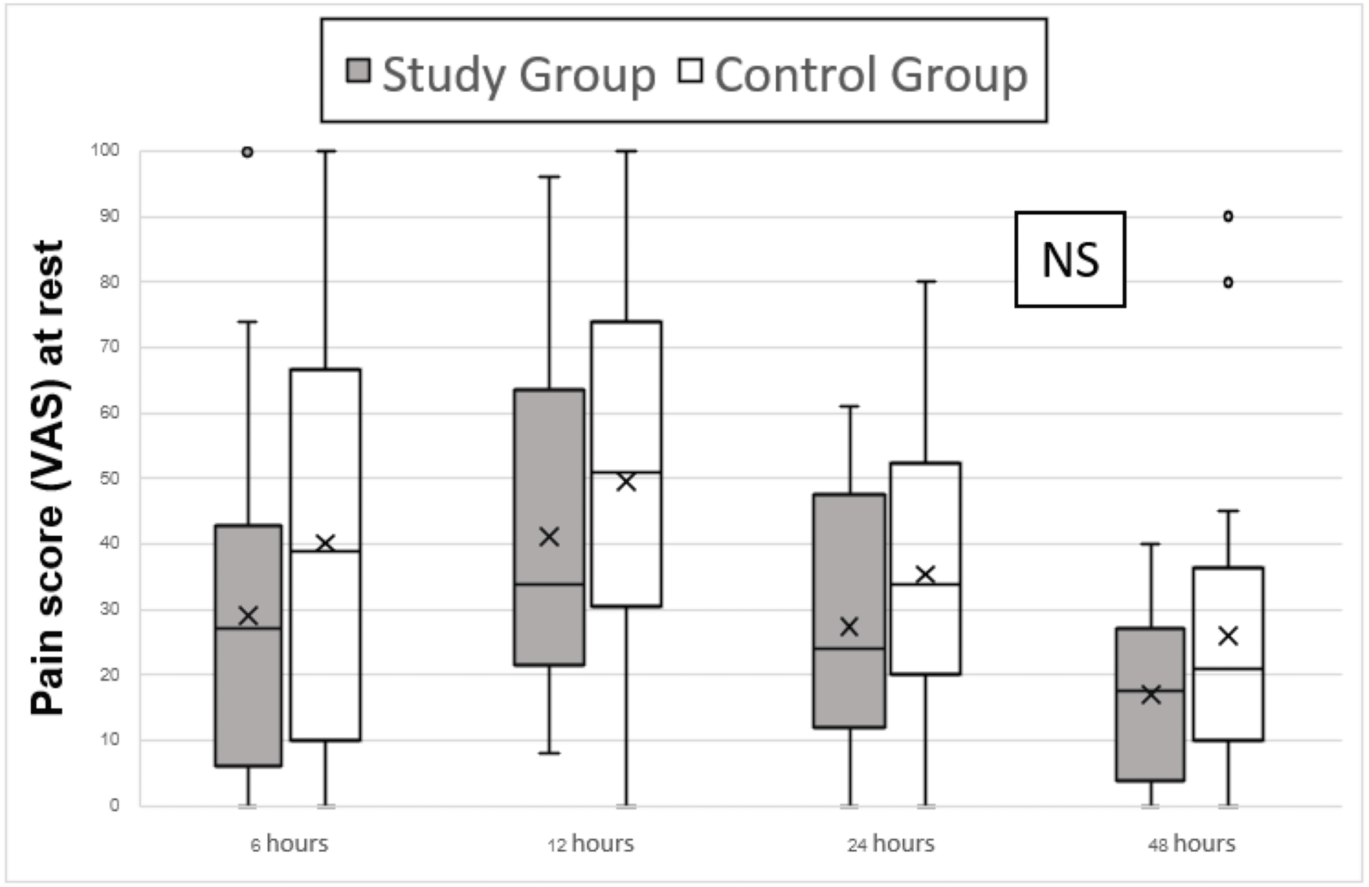
Pain score (VAS) assessment performed at the first 48 hours post operation at rest NS: no statistically significant difference; VAS: visual analog score

**Figure 3:**
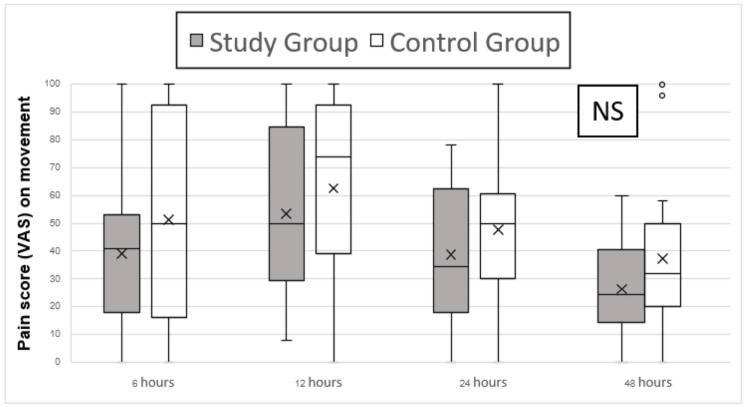
Pain score (VAS) assessment performed first 48 hours post operation on movement NS: no statistically significant difference; VAS: visual analog score

Regarding the primary outcome, the comparison of median pain scores between the two groups at six hours post operation did not reveal any statistically significant differences at rest (p=0.248) and on movement by doing knee flexion at 30 degrees (p=0.253). Nevertheless, it is notable that there was a consistent trend between the groups in both pain score (VAS) at rest (p=0.114) and pain score (VAS) on movement (p=0.131). The peak pain scores were observed at 12 hours post operation, with pain scores at rest exceeding 70 for 11 out of 44 patients (0.25%) and pain scores during movement exceeding 70 for 19 out of 44 patients (43.1%) (Figures 2-3).

**Figure 4:**
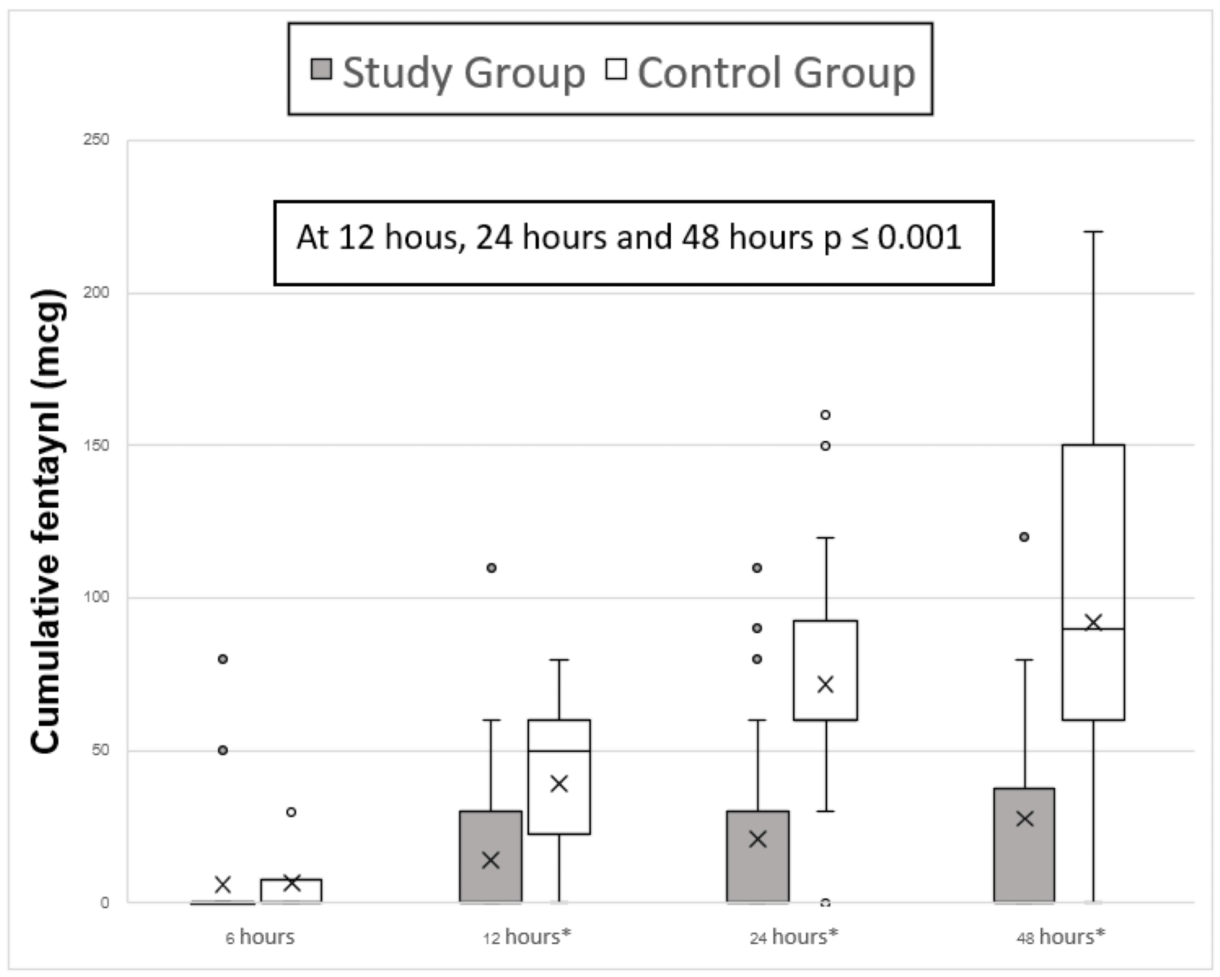
Postoperative fentanyl consumption (mcg) at the first 48 hours post operation (*) = statistically significant difference

For the secondary outcome, comparing median pain scores between the two groups at 12, 24, and 48 hours post operation, both at rest and during knee flexion at 30 degrees, did not show any statistically significant differences (Figures 2-3). However, opioid consumption was significantly lower in the study group at these time points. The study group had median opioid consumption values (interquartile range (IQR)) of 0 (0, 30) at 12, 24, and 48 hours post operation, while the control group had higher consumption with median values (IQR) of 50 (30, 60) at 12 hours, 60 (60, 90) at 24 hours, and 90 (60, 150) at 48 hours post operation (p≤0.001). There was no difference in opioid consumption at six hours post operation (p = 0.301), with both groups showing median values (IQR) of 0 (0, 0) (Figure 4).

This study found that the quadriceps strength at six hours post operation, three patients (13.6%) in the study group and five patients (22.7%) in the control group were unable to elevate their knees, but there is no statistical difference (p=0.434). Similarly, at 12 hours post operation, two patients (9.1%) in the study group and four patients (18.2%) in the control group were unable to perform knee elevation, but there is no statistical difference also (p=0.38).

The incidence of PONV was compared between both groups, with no statistically significant differences observed. Additionally, there were no reports of abnormal ankle movement in either group or LAST event. Notably, there is one patient in the control group who reported itching, affecting one individual (4.5%) at the 24-hour mark post operation. Moreover, no statistically significant differences were noted regarding the most painful quadrant of the knee between the two groups. Similarly, within each group, no significant correlation was observed between the quadrant of knee pain and the surgical procedure (Table 2).

**Table 2:** Secondary outcomes Quadriceps muscle strength was evaluated using straight leg raising for motor grade V; Ankle movement was evaluated via dorsiflexion for motor grade V; Chi-square test and independent t-test or Mann-Whitey U test; A p-value < 0.005 was considered significant hr: hours; PONV: postoperative nausea vomiting; LAST: local anesthetic systemic toxicity

Secondary outcomes	Study group (n=22)	Control group (n=22)	p-value
Quadricep straight, n (%)			
6 hr	19 (86.4%)	17 (77.3%)	0.434
12 hr	20 (90.9%)	18 (81.8%)	0.38
24 hr	22 (100%)	22 (100%)	N/A
48 hr	22 (100%)	22 (100%)	N/A
Ankle movement, n (%)			
6 hr	22 (100%)	22 (100%)	N/A
12 hr	22 (100%)	22 (100%)	N/A
24 hr	22 (100%)	22 (100%)	N/A
48 hr	22 (100%)	22 (100%)	N/A
PONV, n (%)			
6 hr	0 (0%)	1 (4.5%)	0.312
12 hr	1 (4.5%)	2 (9.1%)	0.55
24 hr	1 (4.5%)	2 (9.1%)	0.55
48 hr	0 (0%)	0 (0%)	N/A
Itching, n (%)			
6 hr	0 (0%)	0 (0%)	N/A
12 hr	0 (0%)	0 (0%)	N/A
24 hr	0 (0%)	1 (4.5%)	0.312
48 hr	0 (0%)	0 (0%)	N/A
Most painful quadrant, n (%)			
Medial upper knee	7 (31.8%)	4 (18.2%)	0.112
Medial lower knee	13 (59.1%)	11 (50%)	-
Lateral upper knee	0 (0%)	5 (22.7%)	-
Lateral lower knee	2 (9.1%)	2 (9.1%)	-
LAST event	0 (0%)	0 (0%)	N/A

## Discussion

In our study, we found that there were no statistically significant differences observed in median pain scores at any recorded time point, especially at six hours post operation which we hypothesized. However, the average median pain scores in the study group still appeared consistently lower across all recorded time points post operation compared to the control group.

The study group showed a statistically significant reduction in postoperative opioid consumption, with median values (IQR) of 0 (0, 30) at 12 hours post operation, compared to the control group (p≤0.001). However, there was no reduction in opioid use at six hours post operation, likely due to the lingering effects of spinal anesthetic analgesia. This suggests that adding GNB to ACB reduced opioid consumption during early recovery, thereby promoting better ambulation.

There were no statistically significant differences in quadriceps strength between the GNBs plus ACB group and the ACB alone group. The inability of certain patients to elevate their knees was not attributed to the treatment intervention but rather to the overarching presence of excessive pain hindering knee elevation.

Additionally, there were no statistically significant differences between the two groups in the incidence of side effects such as PONV, itching, abnormal ankle movement, or LAST. Our analysis also found no significant differences between the treatment groups regarding the quadrant of knee pain. Similarly, within each group, there was no significant correlation between the quadrant of knee pain and the specific surgical procedure performed.

A comprehensive understanding of knee surgical anatomy and innervation involves the selection of an appropriate regional anesthesia technique in clinical practice. Various single regional anesthesia techniques exist, offering multimodal analgesia for patients undergoing arthroscopic knee surgery. However, each peripheral nerve block and local infiltration method carries its own advantages and limitations. Consequently, anesthesiologists must carefully evaluate individual patient factors to determine the most suitable peripheral nerve block technique, thereby optimizing outcomes and minimizing patient risk.

The sensory innervation of the knee shows intricate anatomical variability [13-15]. Studies have revealed that the anterior knee capsule receives sensory supply from various regions: the superolateral aspect is innervated by the nerve to vastus lateralis (NVL) and nerve to vastus intermedius (NVI), and the superior gluteal nerve (SLGN) (from either the common peroneal or sciatic nerve). The superomedial region is supplied by the nerve to vastus medialis (NVM), NVI, and superomedial genicular nerve (SMGN) (all femoral nerve branches). The inferolateral aspect receives innervation from the recurrent branch of the common perineal nerve (CPN) and inferolateral genicular nerve (ILGN). The inferomedial aspect is innervated by the infrapatellar branch of the saphenous nerve (SPN) and the inferomedial genicular nerve (IMGN) (a tibial nerve branch). Additionally, the obturator nerve (ON) may provide sensory supply to the inferomedial thigh and anteromedial knee capsule. The posterior knee capsule's sensory supply includes articular branches from the tibial nerve (TN) and the posterior branch of the ON, with minor contributions from the common peroneal and sciatic nerves. The tibial nerve branches supply the entire posterior capsule, with the superomedial aspect also receiving input from the ON, and the superolateral aspect potentially receiving input from the sciatic and common peroneal nerves [16-18].

In our study, the group receiving GNB plus ACB had superolateral, superomedial, and inferomedial genicular blocks. We avoided the inferolateral genicular block to prevent unintended motor weakness or potential foot drop from blocking the common peroneal nerve. The mid-thigh ACB targets the saphenous nerve, medial cutaneous FN, NVM, and possibly the ON's articular branches. While an ACB is popular for its motor-sparing analgesic effects, it doesn't provide full knee blockade [19-21]. However, combining an ACB with GNBs shows promise for extensive knee coverage.

Limitations

The limitations of the study include the following: first is a restricted sample size, potentially impeding accurate effect estimation. Second, the complex innervation of the knee and the variability in anatomical planes through which nerves traverse could account for the inconsistency in block results. However, this aspect couldn't be evaluated due to the sequential administration of peripheral nerve block after spinal anesthetic block. Thirdly, we did not have a control group that did not receive any blocks in this study. However, conducting an ACB would likely be more beneficial than administering no blocks. Thus, we posit that while we did not specifically examine patients without an ACB in this study, it is highly probable that such patients would exhibit higher pain scores and consume larger amounts of opioids postoperatively. Additionally, for ethical reasons, we aimed to save the number of patients enrolled in the study. Furthermore, it is advisable to utilize intravenous patient control analgesia (IV-PCA) instead of intermittent opioid IV bolus for assessing opioid consumption.

## Conclusions

Based on the results of this study, we conclude that adding GNBs to an ACB does not reduce pain in patients undergoing arthroscopic knee surgery, although it did reduce opioid consumption significantly between 12 to 48 hours post operation. The peak time for pain occurred 12 hours post operation, highlighting the need for better pain management during this period. The ultrasound-guided GNBs, performed with the patient in a supine position, are a straightforward technique. Additionally, there were no significant side effects from combining GNBs with an ACB, including quadriceps motor block, nausea, vomiting, itching, or LAST, indicating a good safety profile for this approach.

## References

[1] Howard DH (2018). Trends in the use of knee arthroscopy in adults. JAMA Intern Med.

[2] Drosos GI, Stavropoulos NI, Katsis A, Kesidis K, Kazakos K, Verettas DA (2008). Post-operative pain after knee arthroscopy and related factors. Open Orthop J.

[3] Jenstrup MT, Jæger P, Lund J (2012). Effects of adductor-canal-blockade on pain and ambulation after total knee arthroplasty: a randomized study. Acta Anaesthesiol Scand.

[4] Hanson NA, Derby RE, Auyong DB (2013). Ultrasound-guided adductor canal block for arthroscopic medial meniscectomy: a randomized, double-blind trial. Can J Anaesth.

[5] Konya ZY, Akin Takmaz S, Başar H, Baltaci B, Babaoğlu G (2020). Results of genicular nerve ablation by radiofrequency in osteoarthritis-related chronic refractory knee pain. Turk J Med Sci.

[6] Sinha C, Singh AK, Kumar A, Kumar A, Kumar S, Kumari P (2022). Analgesic effect of continuous adductor canal block versus continuous femoral nerve block for knee arthroscopic surgery: a randomized trial. Braz J Anesthesiol.

[7] Vijay M (2020). Comparing continuous adductor canal block alone, with combined continuous adductor canal block with ipack in terms of early recovery and ambulation in patients undergoing unilateral total knee replacement- a prospective randomized double blinded study. J Evid Based Med.

[8] Tak R, Gurava Reddy AV, Jhakotia K, Karumuri K, Sankineani SR (2022). Continuous adductor canal block is superior to adductor canal block alone or adductor canal block combined with IPACK block (interspace between the popliteal artery and the posterior capsule of knee) in postoperative analgesia and ambulation following total knee arthroplasty: randomized control trial. Musculoskelet Surg.

[9] Rambhia M, Chen A, Kumar AH, Bullock WM, Bolognesi M, Gadsden J (2021). Ultrasound-guided genicular nerve blocks following total knee arthroplasty: a randomized, double-blind, placebo-controlled trial. Reg Anesth Pain Med.

[10] Cuñat T, Mejía J, Tatjer I (2023). Ultrasound-guided genicular nerves block vs. local infiltration analgesia for total knee arthroplasty: a randomised controlled non-inferiority trial. Anaesthesia.

[11] Rosner B (2000). Fundamentals of Biostatistics. 5th Edition. Fundamentals of Biostatistics. 5th ed. Duxbury: Thomson Learning.

[12] Ngamjarus C, Chongsuvivatwong V (2014). n4Studies: Sample Size and Power Calculations for iOS.

[13] Kukreja P, Venter A, Mason L (2021). Comparison of genicular nerve block in combination with adductor canal block in both primary and revision total knee arthroplasty: a retrospective case series. Cureus.

[14] Cankurtaran D, Karaahmet OZ, Yildiz SY, Eksioglu E, Dulgeroglu D, Unlu E (2020). Comparing the effectiveness of ultrasound guided versus blind genicular nerve block on pain, muscle strength with isokinetic device, physical function and quality of life in chronic knee osteoarthritis: a prospective randomized controlled study. Korean J Pain.

[15] Horner G, Dellon AL (1994). Innervation of the human knee joint and implications for surgery. Clin Orthop Relat Res.

[16] Tran J, Peng PW, Gofeld M, Chan V, Agur AM (2019). Anatomical study of the innervation of posterior knee joint capsule: implication for image-guided intervention. Reg Anesth Pain Med.

[17] Franco CD, Buvanendran A, Petersohn JD, Menzies RD, Menzies LP (2015). Innervation of the anterior capsule of the human knee: implications for radiofrequency ablation. Reg Anesth Pain Med.

[18] Tran J, Peng PW, Lam K, Baig E, Agur AM, Gofeld M (2018). Anatomical study of the innervation of anterior knee joint capsule: implication for imageguided intervention. Reg Anesth Pain Med.

[19] Jeng CL, Torrillo TM, Rosenblatt MA (2010). Complications of peripheral nerve blocks. Br J Anaesth.

[20] Bendtsen TF, Moriggl B, Chan V, Børglum J (2016). The optimal analgesic block for total knee arthroplasty. Reg Anesth Pain Med.

[21] Egeler C, Jayakumar A, Ford S (2013). Motor-sparing knee block - description of a new technique. Anaesthesia.

